# High Sodium-Induced Oxidative Stress and Poor Anticrystallization Defense Aggravate Calcium Oxalate Crystal Formation in Rat Hyperoxaluric Kidneys

**DOI:** 10.1371/journal.pone.0134764

**Published:** 2015-08-04

**Authors:** Ho-Shiang Huang, Ming-Chieh Ma

**Affiliations:** 1 Department of Urology, National Cheng Kung University Hospital, Tainan, Taiwan; 2 School of Medicine, Fu Jen Catholic University, New Taipei, Taiwan; University of São Paulo School of Medicine, BRAZIL

## Abstract

Enhanced sodium excretion is associated with intrarenal oxidative stress. The present study evaluated whether oxidative stress caused by high sodium (HS) may be involved in calcium oxalate crystal formation. Male rats were fed a sodium-depleted diet. Normal-sodium and HS diets were achieved by providing drinking water containing 0.3% and 3% NaCl, respectively. Rats were fed a sodium-depleted diet with 5% hydroxyl-L-proline (HP) for 7 and 42 days to induce hyperoxaluria and/or calcium oxalate deposition. Compared to normal sodium, HS slightly increased calcium excretion despite diuresis; however, the result did not reach statistical significance. HS did not affect the hyperoxaluria, hypocalciuria or supersaturation caused by HP; however, it increased calcium oxalate crystal deposition soon after 7 days of co-treatment. Massive calcium oxalate formation and calcium crystal excretion in HS+HP rats were seen after 42 days of treatment. HP-mediated hypocitraturia was further exacerbated by HS. Moreover, HS aggravated HP-induced renal injury and tubular damage via increased apoptosis and oxidative stress. Increased urinary malondialdehyde excretion, *in situ* superoxide production, NAD(P)H oxidase and xanthine oxidase expression and activity, and decreased antioxidant enzyme expression or activity in the HS+HP kidney indicated exaggerated oxidative stress. Interestingly, this redox imbalance was associated with reduced renal osteopontin and Tamm-Horsfall protein expression (via increased excretion) and sodium-dependent dicarboxylate cotransporter NaDC-1 upregulation. Collectively, our results demonstrate that a HS diet induces massive crystal formation in the hyperoxaluric kidney; this is not due to increased urinary calcium excretion but is related to oxidative injury and loss of anticrystallization defense.

## Introduction

Sodium restriction is a common recommendation to patients with kidney stones because high sodium (HS) intake is a major dietary risk factor for stone formation [1, 2]. HS intake increases the urinary excretion of calcium, in part via reduced passive reabsorption of calcium following that of sodium and water in tubules [1, 3]. Excessive urinary calcium excretion is part of the etiology of calcium-containing stone formation and contributes to the supersaturation of calcium oxalate (CaOx) crystals [4]. Moreover, HS diet may also produce an undesirable reduction in urinary citrate excretion via an uncertain mechanism [5]. Hypercalciuria and hypocitraturia are therefore thought to mediate stone formation upon HS intake [1–3, 5]. After glomerular filtration, citrate reabsorption mainly occurs in the proximal tubule via sodium-dependent dicarboxylate cotransporter-1 (NaDC-1) [6]. Immunohistochemical staining demonstrated that NaDC-1 upregulation in the stone-forming kidneys of nephrolithiasis patients and in the kidneys of rats with metabolic acidosis [7, 8]. Since NaDC-1 works as a sodium-dependent transport protein, it is unclear whether HS has an effect on NaDC-1 in terms of calcium crystal formation.

HS intake induces intrarenal oxidative stress via increased NAD(P)H oxidase (NOX) activity and decreased expression of superoxide dismutase (SOD) [9]. Interestingly, oxidative stress plays a critical role in the pathogenesis of CaOx crystal formation in hyperoxaluric kidneys [10–13]. Exposure of tubular cells and rat kidneys to oxalate increases superoxide production *in vitro* and *in vivo* [11, 13–15]. We previously demonstrated that an imbalance in intrarenal oxidative/antioxidant protein activity contributes to superoxide formation in the rat hyperoxaluric kidney [10]. Oxidative stress causes tubular cells to be more vulnerable when exposed to oxalate or crystals. Once damaged, renal tissues increase their release of cytokines and chemokines, resulting in the attraction and accumulation of inflammatory cells at the site of injury [14, 16, 17]. Consequently, the increased oxidative stress induced by leukocyte infiltration and the lowered self-defense of renal tissues against oxidative injury leads to more tubular cell damage [12, 13]. It is unknown whether oxidative stress induced by HS intake causes more injury to the hyperoxaluric kidney and greater CaOx crystal formation.

The osteopontin (OPN) and Tamm-Horsfall protein (THP) secreted by tubular cells are important molecules to prevent crystal formation [12, 18, 19]. We previously demonstrated that lowered renal expression and increased urinary excretion of OPN and THP are associated with aggravated oxidative stress in rat hyperoxaluric kidneys [12]. THP plays a protective role in ischemic renal injury [20]. Animals lacking THP have more tubular casts, increased inflammation and necrosis, and worse renal function [20]. OPN is important for cell-cell and cell-matrix binding, and downregulation of OPN impairs tubular cell regeneration during recovery from cisplatin-induced renal damage [21]. It is unclear whether HS may affect renal production and excretion of OPN and THP during oxidative stress-mediated CaOx crystal formation.

The present study used hydroxy-L-proline (HP) (which has little effect on urinary pH after catabolism *in vivo*) as a precursor of oxalate to induce hyperoxaluria and/or CaOx crystal formation in rats [17] and to assess the effects of HS. The results revealed that HS itself induced diuresis. The diuretic response to HS however had no effect on the hyperoxaluria or urinary supersaturation caused by HP. HS increased the renal injury caused by hyperoxaluria via tubular cell damage, apoptosis, and redox imbalance-mediated oxidative stress. The effects of HS in hyperoxaluric kidneys were associated with impairment of endogenous antilithic defense via upregulation of NaDC-1 and reduced expression of OPN and THP.

## Materials and Methods

### Experimental animals

Male Wistar rats (BioLASCO, Taipei, Taiwan) weighing 200–210 g were used in all experiments. All animal experiments were performed in compliance with the Guide for the Care and Use of Laboratory Animals (published by National Academy Press, Washington DC, 2011) and were reviewed and approved by the Institutional Animal Care and Use Committee (permit number: A9919) of the Fu Jen Catholic University in Taiwan. All surgery was performed under sodium pentobarbital anesthesia, and all efforts were made to minimize suffering.

### Experimental procedures

Rats were housed at constant temperature with a 9 h light/15 h dark cycle (light from 08:00 to 17:00). All rats were fed a sodium-deficient (SD) diet (ICN Biomedicals, Aurora, Ohio) and supplied with 0.3% NaCl (NS) or 3% NaCl (HS) via the drinking water as described previously [3, 22]. The age-matched rats were then divided into groups as follows for 7 or 42 days of induction: (1) the NS rats received a SD diet and 0.3% NaCl in the drinking water; (2) the HP rats were given a SD diet mixed with 5% HP (weight/weight, Sigma, St. Louis, MO) and drinking water containing 0.3% NaCl; (3) the HS rats received a SD diet and 3% NaCl in the drinking water; and (4) the HS+HP rats received a SD diet mixed with 5% HP and drinking water containing 3% NaCl. Six rats were included in each group. During the experimental period, all rats had free access to chow and drinking water. The rats were placed in individual metabolic cages for 2 days and acclimated before collection of 24-h urine samples for urinalysis.

### Urinalysis and urine biochemistry

After adaptation in the metabolic cage, urine samples were collected for 24 h into tubes containing penicillin G (2000 IU) and streptomycin (2000 IU) to prevent microbial overgrowth. Food and water intake were also determined. Urine output was expressed in terms of ml/day. Briefly, urine samples were centrifuged at 620 *g* for 10 min. The supernatant was separated from the sediment and collected for biochemical assays using commercially available ELISA kits. The sediment was then dried for 2–3 days in an oven at 60°C to measure dry weight. Urinary calcium and sodium levels were measured using an electrolyte analyzer (Dri-Chem 3500i, Fuji, Tokyo, Japan) as described previously [23, 24]. The magnesium concentration in the urine was determined using a commercial kit (BioAssay Systems, Hayward, CA). Urinary oxalate, citrate, and creatinine levels were determined as described previously [23, 24]. The degree of urine supersaturation was estimated according to AP(CaOx)index using the formula (4076×calcium^0.9^×oxalate^0.96^)/[(citrate+0.015)^0.60^×magnesium^0.55^×urine volume^0.99^)] as described previously [25]. Urinary NAG (a marker of tubular damage) was measured using a commercial kit as described previously [10]. ELISAs for determining urinary levels of OPN (R&D Systems, Wiesbaden, Germany) and THP (MD Biosciences, St. Paul, MN) were performed according to the protocols provided by suppliers. Oxidative stress was evaluated by measuring urinary MDA levels with a commercial kit (OxisResearch, Portland, OR). Urinalysis was performed on technical replicates.

### Detection of *in situ* superoxide formation in the kidney

After the metabolic cage study, rats were anesthetized with pentobarbital sodium (50 mg/kg) and 200 μl of blood was taken from the abdominal aorta to measure plasma creatinine levels [10, 24]. Creatinine clearance (Ccr) was calculated as the index of renal function in each group. The rats then underwent transcardiac perfusion with 500 ml of phosphate-buffered saline (pH 7.4) as described previously [10, 12, 24]. The right kidney was removed and stored at -80°C for further analysis of protein expression and activity. Briefly, after right nephrectomy, the left kidney was perfused with 10 μM of DHE (Sigma) solution at 4°C (rate, 1 ml/min) for 30 min to determine in situ superoxide production in the kidney. DHE can enter cells and be oxidized by superoxide to yield ethidium, which binds DNA and produces bright red fluorescence. An increase in ethidium-DNA fluorescence indicates endogenous superoxide production within cells [26]. The perfused kidney was then embedded in O.C.T., and 10-μm sections prepared. Fluorescence intensity was examined under an inverted fluorescence microscope.

### CaOx crystal deposition and crystalluria

Tissue slices were prepared for staining with hematoxylin and eosin and examined by polarizing microscopy (model BH-2, Olympus, Tokyo, Japan) for the presence of CaOx crystals as described previously [23, 24]. The total amount of CaOx crystal formation was assumed to be equal to the crystal deposition in the kidney plus crystals found in the urinary bladder and in a 24 h urinary specimen. In brief, the extent of CaOx crystal deposition was graded semiquantitatively as follows: “no crystal deposit” (grade 0) to “massive crystal deposits” (grade 3).

### Measurement of oxidant and antioxidant enzyme activity

The enzymatic activity of Cu/ZnSOD, MnSOD, GPx, and catalase (CAT) in the kidneys was measured using commercial kits (Oxis International) as described previously [10]. NADH (0.1 mmol/L) and xanthine (0.1 mmol/L) were used as substrates for NOX and XO, respectively. Salmon testis DNA was added to the reaction mixture to stabilize ethidium fluorescence and increase the sensitivity of the assay [26]. Enzyme activity is presented as fluorescence units/10 sec/mg protein.

### Western blot analysis

The protein expression of gp91phox-containing NOX, XO, CAT, GPx, CuZnSOD, MnSOD, OPN, THP, NaDC-1, cytochrome c (an indication of apoptosis), and full-length and cleaved PARP (an indicator of the DNA damage response to oxidative injury) were examined by immunoblot analysis as described previously [23, 24, 26]. Primary antibodies to catalase, GPx, CuZnSOD, and MnSOD were purchased from The Binding Site (diluted 1:1000; Birmingham, England, UK). Primary antibodies to gp91phox, XO, cytochrome c (cyto c), PARP, OPN, THP, NaDC-1, and actin were obtained from Santa Cruz Biotechnology (diluted 1:2000; Santa Cruz, CA). In brief, the same amount of protein from each preparation was separated on SDS polyacrylamide gels under denaturing conditions and electrophoretically transferred to a polyvinylidene difluoride membrane (Amersham-Pharmacia Biotech, Little Chalfont, UK). The membrane was incubated with the appropriate primary antibody overnight at 4°C. After washing, the membrane was incubated for 1 h at room temperature with the corresponding secondary antibody conjugated to horseradish peroxidase (1:200; Vector Laboratories, Burlingame, CA). Bound antibody was visualized with a commercial enhanced chemiluminescence kit (Amersham-Pharmacia Biotech) and Kodak film. Band density was measured semiquantitatively using an image analytical system (Diagnostic Instruments, Sterling Heights, MI). The level of each protein was expressed relative to the amount of actin.

### Detection of redox enzyme mRNA expression by real-time RT-PCR

Real-time PCR was performed in an ABI StepOne Plus system (Applied Biosystems Foster City, CA) as described previously [27]. The reaction mixture (20 μL total volume) comprised 200 ng of cDNA, 30 μmol of each primer (Table 1), and Sybr Green (PCR master mix kit; Applied Biosystems). The thermal cycling conditions were as follows: initial denaturation at 95°C for 20 s, followed by 40 cycles at 95°C for 1 s and 60°C for 20 s. Melting curve analysis was performed at the end of each PCR experiment. All reactions were run in duplicate. Relative changes in gene expression were calculated using the ΔCt (threshold cycle) method, i.e., the raw Ct value of the house-keeping gene (GAPDH) was subtracted from the raw Ct value of the target gene. Changes in target gene expression were calculated using the formula 2^-ΔCt^ and expressed as the fold change relative to the values in control (NS) kidneys.

**Table 1:** Nucleotide sequences of the primers used for real-time quantitative PCR.

Target gene: Product size (Accession number)	Nucleotide sequence (5’-3’)
gp91phox: 155 bp (NM_023965)	Forward: CCAGTGAAGATGTGTTCAGCT
	Reverse: GCACAGCCAGTAGAAGTAGAT
XO: 126 bp (NM_017154)	Forward: GCTTGAATCCTGCCATTGAT
	Reverse: AGGTACVGGTGCCACGAGTA
Cu/ZnSOD: 192 bp (NM_017050)	Forward: GTTCCGAGGCCGCCGCGCGT
	Reverse: GTCCCCATATTGATGGAC
MnSOD: 258 bp (NM_017051)	Forward: CTGAGGAGAGCAGCGGTCGT
	Reverse: CTTGGCCAGCGCCTCGTGGT
Catalase; 116 bp (NM_012520)	Forward: GGCAGCTATGTGAGAGCC
	Reverse: CTGACGTCCACCCTGACT
GPx: 290 bp (NM_030826)	Forward: CTCTCCGCGGTGGCACAGT
	Reverse: CCACCACCGGGTCGGACATAC

Gp91phox, as for NADPH oxidase; XO, xanthine oxidase; Cu/ZnSOD, copper and zinc superoxide dismutase; MnSOD, manganese superoxide dismutase; GPx, glutathione peroxidase.

### Statistical analysis

Statistical analysis was conducted using SPSS 15.0. Differences between subgroups were analyzed using an unpaired *t*-test or one-way ANOVA, while Duncan’s multiple-range test was used to compare subgroups. Differences were regarded as significant at *P* < 0.05.

## Results

### Effect of HS and hyperoxaluric diets on basic health parameters

**Fig 1:**
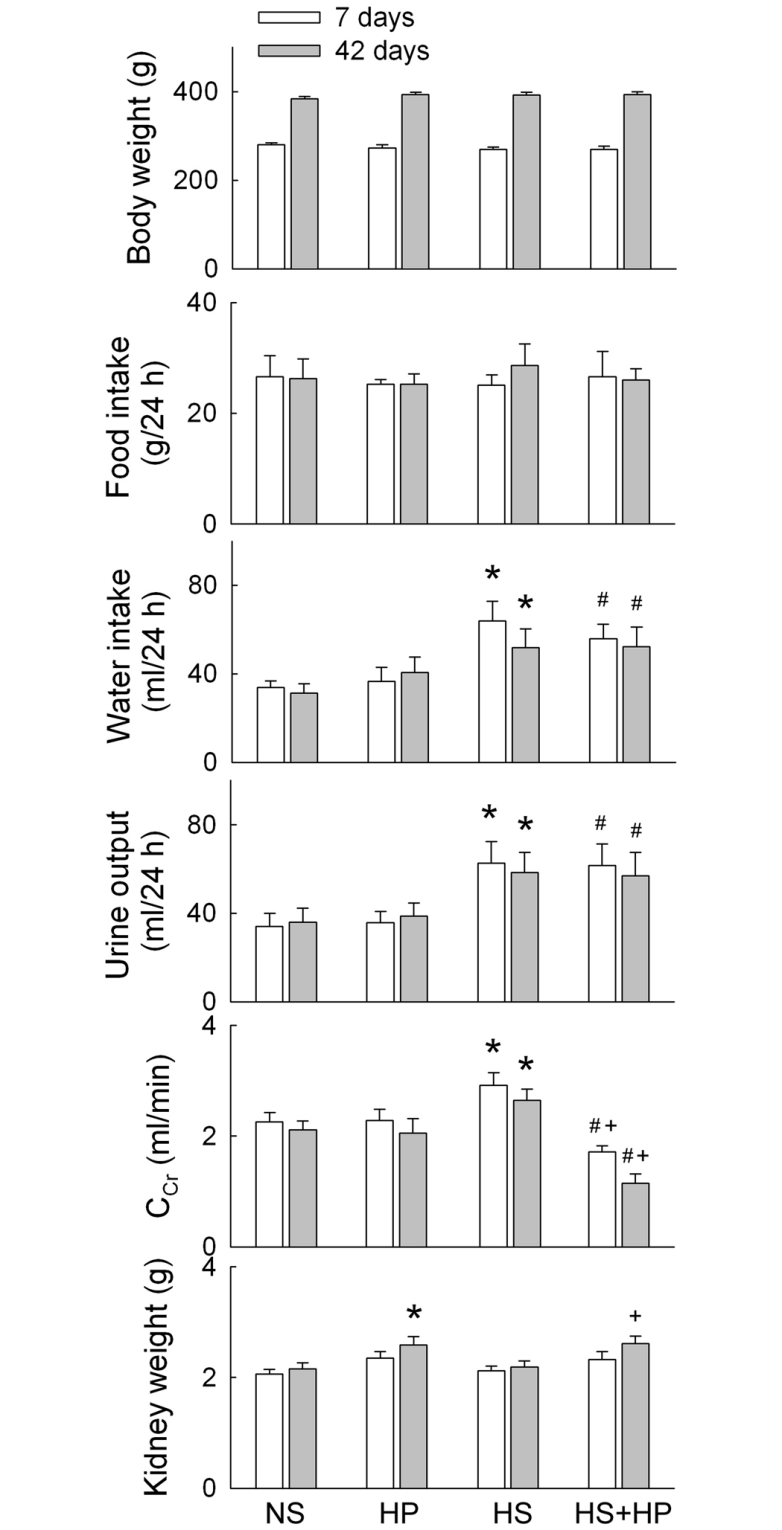
Basic health parameters and renal function. Data were obtained from the metabolic cage study (collected over a 24 h period on Days 7 and 42). N = 6 in each group and time-point. Ccr, creatinine clearance; NS, normal sodium (0.3% NaCl in drinking water); HP, rats treated with 5% hydroxyl-L-proline; HS, high sodium (3% NaCl in drinking water). *p<0.05, HP or HS *vs*. NS group; #p<0.05, HS+HP *vs*. HP group; +p<0.05, HS+HP *vs*. HS group.

There was no difference in body weight or amount of food intake between groups (Fig 1). Compared to the normal sodium (NS) group, the HS group consumed more drinking solution (p<0.05) and had a greater urine output (p<0.05). HP did not affect water intake or urine output when combined with HS. Compared with NS, HP did not affect renal function (creatinine clearance) after 7 or 42 days of treatment. HS itself increased creatinine clearance at both time points, possibly due to increased urine output. Renal insufficiency, measured by low creatinine clearance, occurred in the HS+HP rats at both time points. The weight of both kidneys in the HP group was significantly increased after 42 days of treatment, and combined treatment with HS did not affect this result.

### Effect of HS and hyperoxaluric diets on urinary supersaturation

**Fig 2:**
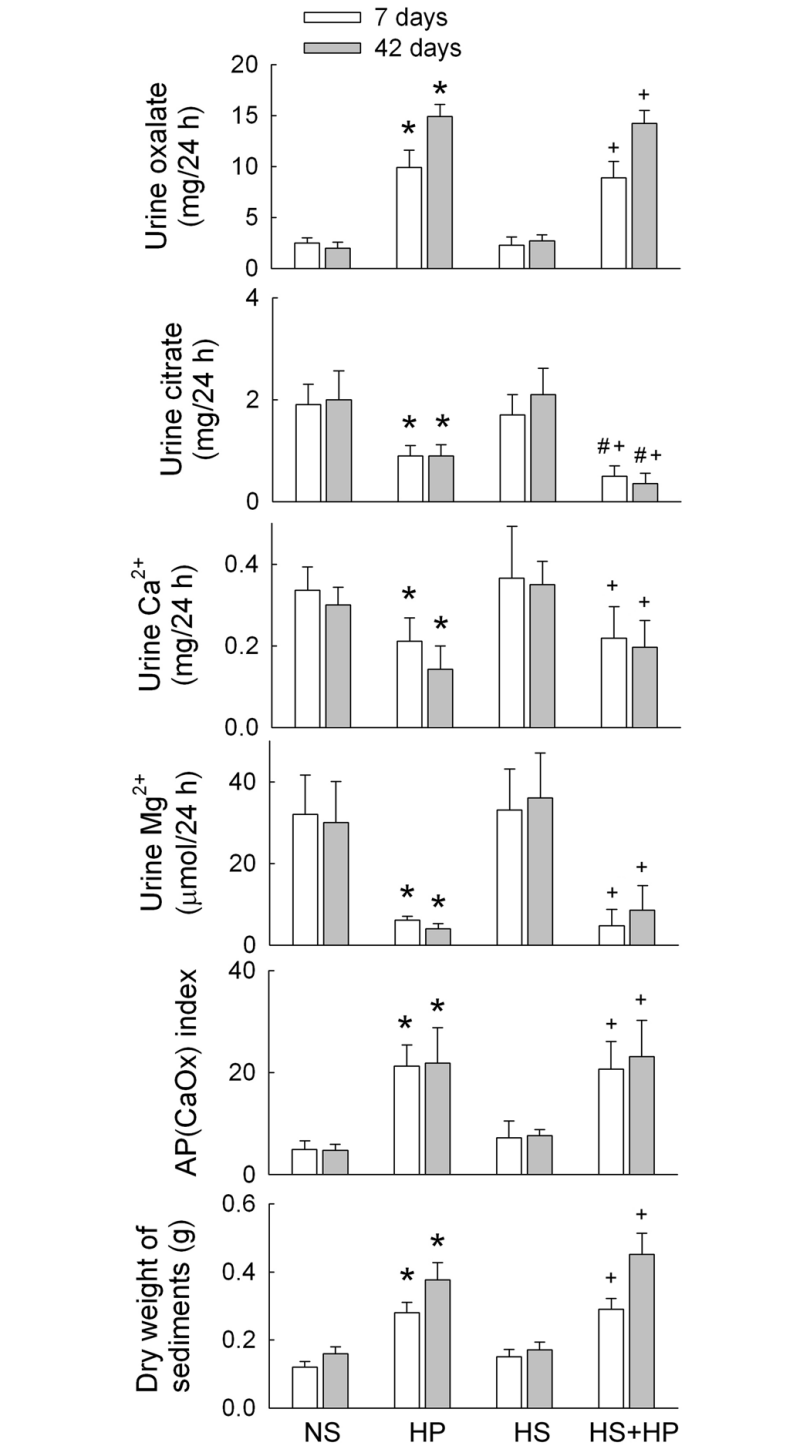
Urinalysis, supersaturation, and urine sediments. Urine was collected from metabolic cages (over a 24 h period on Days 7 and 42) and analyzed for oxalate, citrate, and various ions to estimate supersaturation according to the AP(CaOx)index. The precipitated particles in urine were dried to determine the weight of urine sediments. N = 6 in each group and time-point. NS, normal sodium; HP, hydroxyl-L-proline; HS, high sodium. *p<0.05, HP or HS *vs*. NS group; #p<0.05, HS+HP *vs*. HP group; +p<0.05, HS+HP *vs*. HS group.

Hyperoxaluria, hypocitraturia, hypocalciuria, and hypomagnesuria were seen in rats fed with HP compared to those supplied with NS (Fig 2). HS alone slightly increased urinary calcium excretion at both time points, although the result did not reach statistical significance; however, it had no effect on the urinary excretion of oxalate, citrate, or magnesium. HS did not affect HP-induced hyperoxaluria, hypocalciuria, or hypomagnesuria, but further lowered the urinary excretion of citrate at Days 7 and 42.

Compared to NS, HP significantly increased supersaturation in the urine, as measured by an increased the ion activity product of CaOx [AP(CaOx)index]. HS alone had no effect on AP(CaOx)index. The increased supersaturation seen in HP rats was not affected by HS when HP and HS were co-administered.

We previously showed that massive CaOx crystals could be found in urine sediments in rats fed a hyperoxaluric diet [12]. In the present study, the dry weight of urine sediment increased in the HP group over time was comparable with that in the NS group. HS had no effect on this when given alone or in combination with HP. When assessed by polarizing microscopy, the urine sediments of the HP and HS+HP group were rich in CaOx, especially after 42 days of treatment.

### HS intake increased CaOx formation in the hyperoxaluric kidney

**Fig 3:**
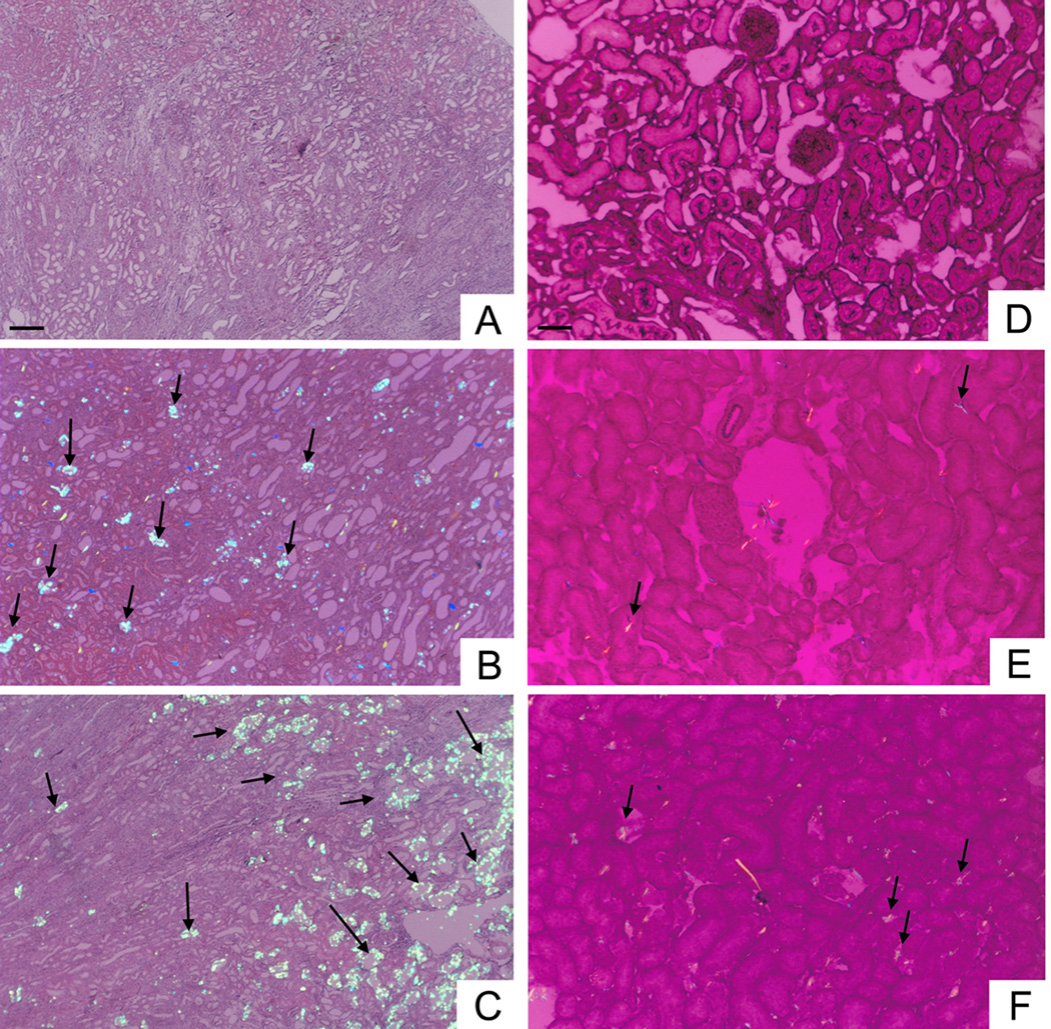
Effect of high sodium and hyperoxaluria on CaOx crystal deposition in kidneys. Left panels (**A–C**) show representative micrographs (obtained by polarizing microscopy) taken after 42 days of treatment: CaOx crystal deposition in the kidneys is indicated by black arrows. Right panels **(D–F)** show kidneys at 7 days. **(A** and **D)** normal sodium (NS) kidney; **(B** and **E)** hyperoxaluric (HP) kidney; **(C** and **F)** high sodium hyperoxaluric (HS+HP) kidney. No CaOx crystals were found in the kidneys of rats treated with HS alone for 7 or 42 days (data not shown). Micrographs **A–C** were taken under a reduction from 10× magnification and micrographs **D–F** were reduced from 40× magnification. Horizontal bars in **A–C** and **D–F** are 500 and 100 μm, respectively.

Increases in the AP(CaOx)index and in the dry weight of urine sediments in HP-treated rats indicated that the intrarenal microenvironment predisposes to *de novo* CaOx formation. Polarizing microscopy revealed no crystals in NS (Fig 3A and 3D) or HS (data not shown) kidneys. In the HP kidney, CaOx crystal deposition of grade 0–1 was seen after 7 days of treatment and grade 1–2 was seen after 42 days (Fig 3E and 3B). In the HS+HP kidney, grade 1–2 CaOx crystal deposition was found after 7 days and grade 3 was seen after 42 days (Fig 3F and 3C). The intrarenal distribution of CaOx crystals between the HP and HS+HP groups was different. In the HP kidney, CaOx crystals were small and deposited evenly in both the renal cortex and the medulla. By contrast, crystals in the HS+HP kidney were large and were deposited predominantly in the renal medulla.

### Effect of HS and hyperoxaluria on tubular damage and oxidative stress

**Fig 4:**
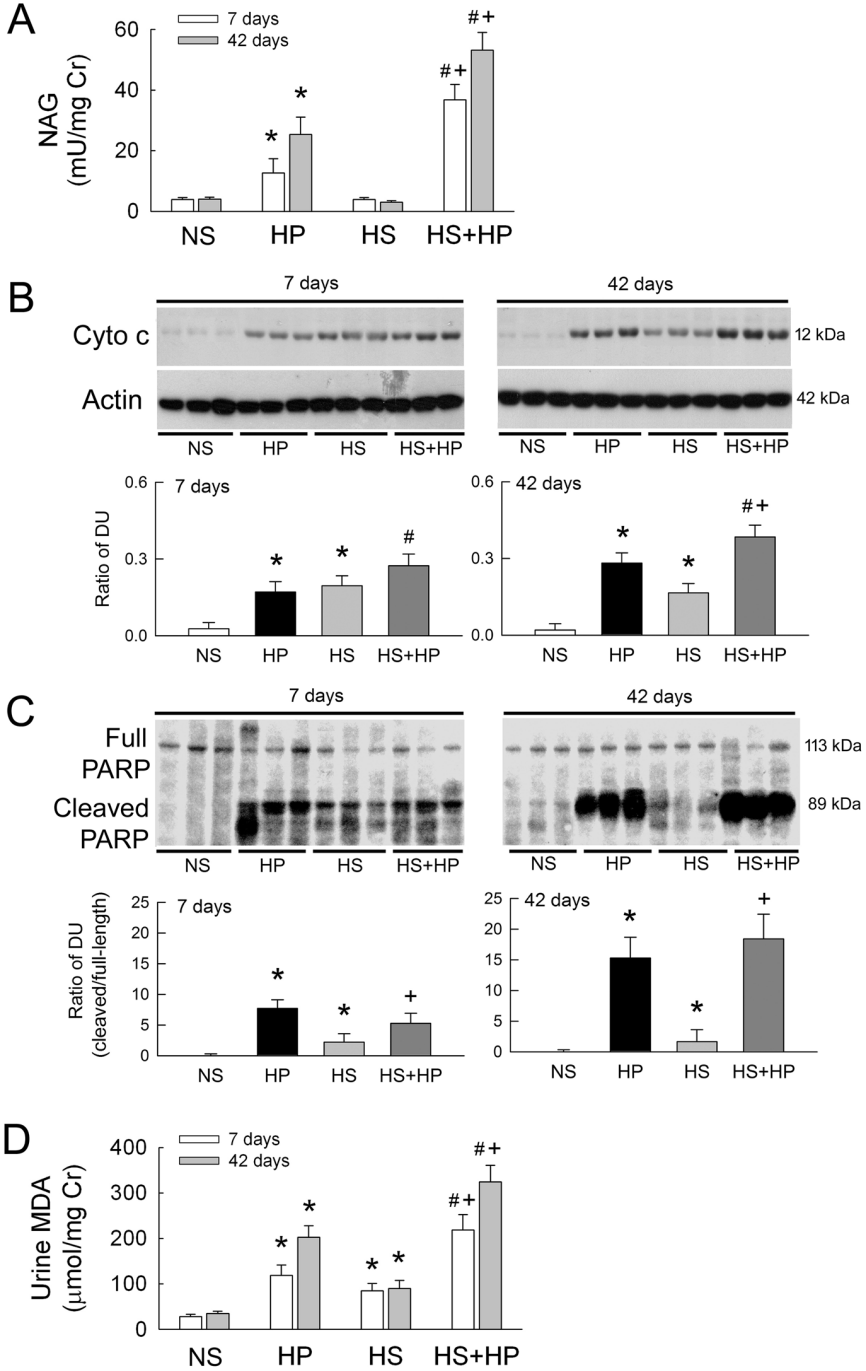
Renal damage makers and lipid peroxidation after high sodium or hyperoxaluric treatment. (**A**) Urinary levels of the tubular enzyme N-acetyl-β-glucosaminidase (NAG) were used as a marker of tubular damage at days 7 and 42. (**B** and **C**) Renal injury was evaluated according to renal expression of cytochrome c (Cyto c) and poly (ADP-ribose) polymerase (PARP), which promote apoptosis after tissue injury. Representative blots from three rats show expression of cytochrome c and full-length and cleaved PARP (40 μg of cytosolic protein per lane). The lower bar graph shows the ratio of cytochrome c to actin, and the ratio of full-length to cleaved PARP as a measure of PARP activity. DU, band density unit. (**D**) Urinary levels of malondialdehyde (MDA) were measured to evaluate intrarenal oxidative stress after 7 and 42 days of HS or HP treatment. N = 6 in each group and time-point. NS, normal sodium; HP, hydroxyl-L-proline; HS, high sodium. *p<0.05, HP or HS *vs*. NS group; #p<0.05, HS+HP *vs*. HP group; +p<0.05, HS+HP *vs*. HS group.

Tubular damage aggregates CaOx crystals [12, 13]. Therefore, we next tested whether HS may affect tubular damage in the HP kidney. Using a sensitive marker of tubular damage, our results showed that urinary excretion of N-acetyl-β-glucosaminidase (NAG) in HP rats increased significantly in a time-dependent manner compared with that in NS rats (Fig 4A). HS itself had no effect on NAG excretion, but aggravated NAG excretion in HP rats.

High enzymuria clearly indicates that HS exacerbates HP-mediated renal injury. We next examined the renal expression of cytochrome c and poly (ADP-ribose) polymerase (PARP), molecules involved in injury-induced apoptosis, to further strengthen our observations. Compared to NS, HP alone and HS alone significantly increased cytochrome c expression after 7 days of induction (Fig 4B). A similar increase in cytochrome c expression was observed in the HS+HP kidney. After 42 days of treatment, an additive increase in cytochrome c expression was seen in the HS+HP kidney compared to the HP or HS kidney. Using the ratio of cleaved to full-length PARP as a measure of protein activity, we found that PARP activity in the HP and HS kidneys was significantly higher than that in the NS kidneys after 7 and 42 days of treatment (Fig 4C). No additive effect of HS on PARP activity was detected in the HP kidneys.

We then asked whether renal injury was due to oxidative stress mediated by HS or hyperoxaluria. Levels of the lipid peroxidation metabolite, MDA, were significantly higher in the urine of HP rats than in that of NS rats (Fig 4D). Interestingly, HS alone also increased urinary malondialdehyde (MDA) excretion, indicating that HS intake caused oxidative stress. When co-administered with HP, HS increased HP-mediated MDA excretion, and therefore oxidative stress, in an additive manner compared to HP alone.

### HS intake increased superoxide generation *in situ*

**Fig 5:**
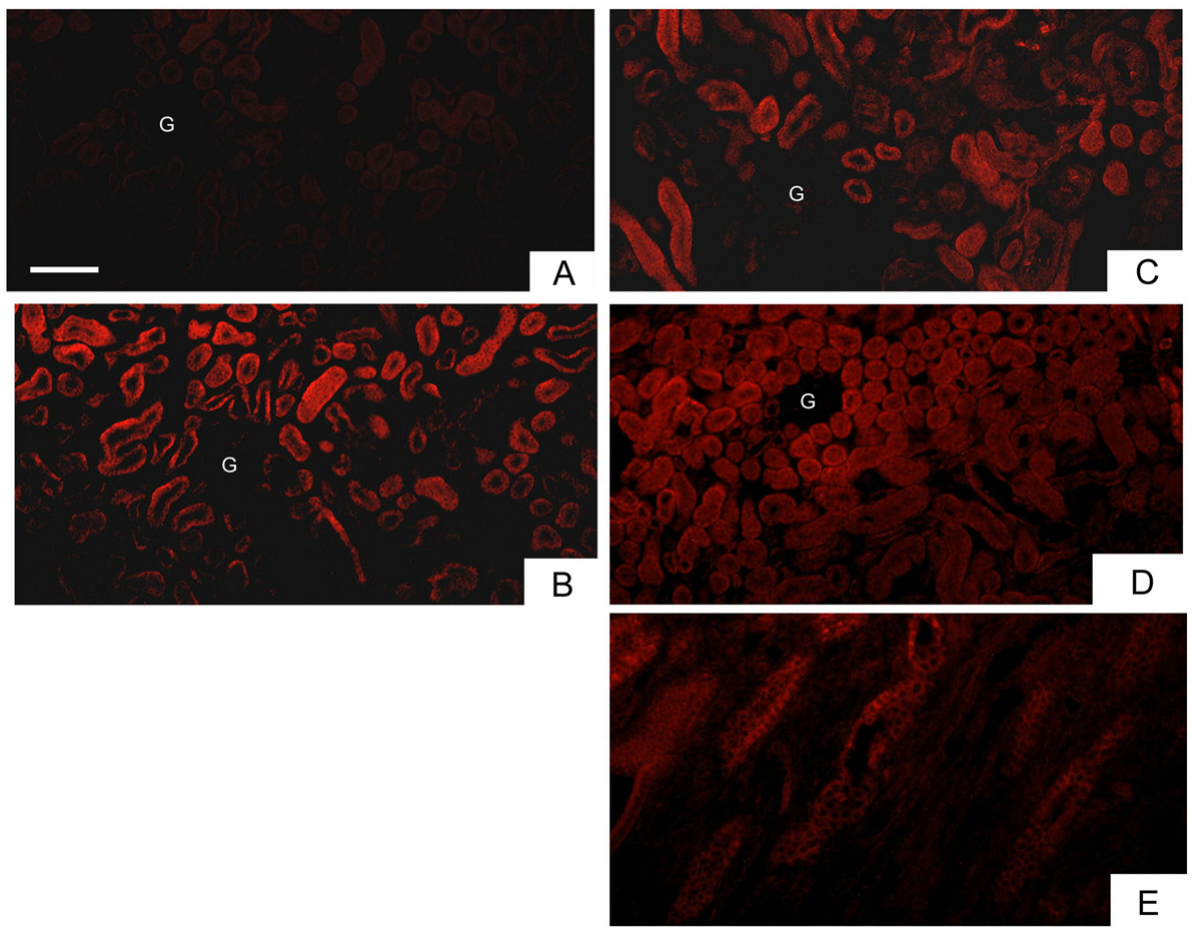
Superoxide production in the kidneys. Kidneys were harvested after 42 days of treatment, perfused with dihydroethidium solution, and immediately frozen in embedding medium. The ethidium-DNA signals, representing increases in superoxide generation, were visualized under an inverted fluorescent microscope. Typical micrographs show superoxide formation in the renal cortex of a normal sodium (NS) kidney (**A**), a hyperoxaluric (HP) kidney (**B**), a high sodium (HS) kidney (**C**), and in the renal cortex (**D**) and renal medulla (**E**) of a HS+HP kidney. G. glomerulus. The horizontal bar represents 200 μm.

Elevation of urinary MDA excretion indicates the possible presence of oxidative stress in the kidneys. We next examined the potential source of renal superoxide generation using dihydroethidium (DHE) staining. At 42 days, superoxide was detected in the distal tubules of NS-treated kidneys; however, the fluorescent signals were fairly faint (Fig 5A). By contrast, strong DHE fluorescence was detected in the distal tubules of the renal cortex in HP-treated kidneys (Fig 5B). HS intake also increased *in situ* superoxide generation, as measured by DHE fluorescence, in the distal tubules (Fig 5C). The DHE signal intensity in the HS+HP kidneys was similar to that in the HS or HP kidneys (Fig 5D); however, the DHE signals were dispersed throughout the tubular and vascular structures in the renal cortex, and also detected as intracellular signals in the renal tubules of the medulla (Fig 5E).

### Changes in oxidative and antioxidant enzyme expression at protein and mRNA levels

**Fig 6:**
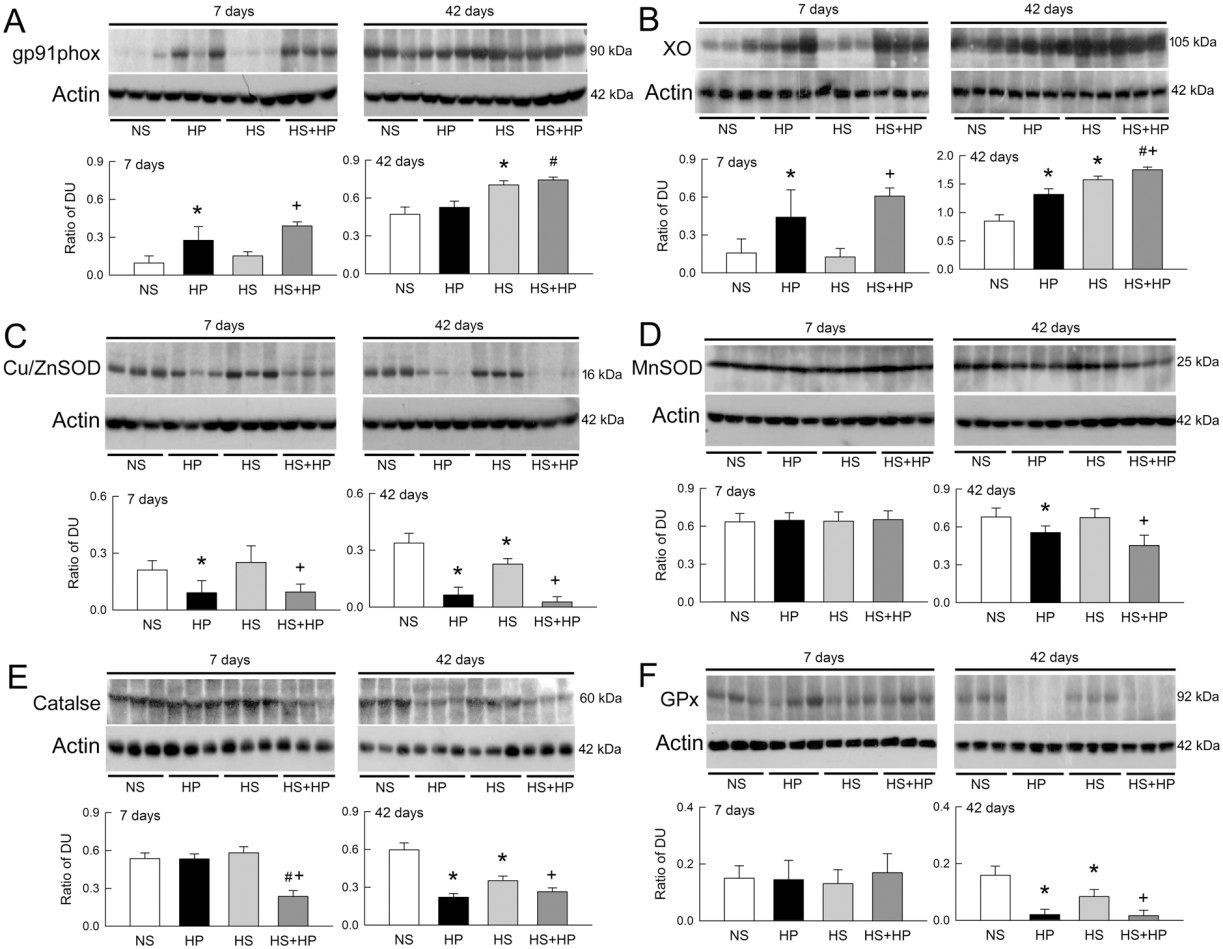
Effect of high sodium and hyperoxaluria on renal expression of oxidative and antioxidant proteins. Western blot analyses of NAD(P)H oxidase (gp91phox) **(A)**, xanthine oxidase (XO) **(B)**, copper and zinc superoxide dismutase (Cu/ZnSOD) **(C)**, manganese superoxide dismutase (MnSOD) **(D)**, catalase **(E)**, and glutathione peroxidase (GPx) **(F)** expression in the kidneys of three representative rats. Equal amounts of protein (40 μg/lane) were loaded. The lower bar graphs show changes in the renal expression of redox proteins, as assessed by densitometry and normalized to actin (n = 6 in each group and time-point). DU, band density unit; NS, normal sodium; HP, hydroxyl-L-proline; HS, high sodium. *p<0.05, HP or HS *vs*. NS group; #p<0.05, HS+HP *vs*. HP group; +p<0.05, HS+HP *vs*. HS group.

Increased superoxide generation indicates the presence of a redox imbalance in the kidneys. Therefore, we next examined the expression of oxidative and antioxidant enzymes to see whether their protein levels were affected by HS or HP. Compared to NS rats, renal expression of gp91phox (a major component of the NOX protein) in HP rats and HS+HP rats increased after 7 days of treatment (Fig 6A). HS, but not HP, increased gp91phox expression at 42 days. An increase in gp91phox expression was seen in HS+HP kidneys at 42 days. HP and HS+HP significantly increased the renal expression of xanthine oxidase (XO) after 7 days compared with that in controls (Fig 6B). XO was also upregulated in HS kidneys after 42 days. Compared to HS alone or HP alone, HS and HP acted synergistically to increase XO.

Compared with that in controls, copper/zinc superoxide dismutase (Cu/ZnSOD) expression decreased in HP and HS+HP kidneys after 7 and 42 days of treatment. Cu/ZnSOD expression was also lowered in the 42 days of HS kidneys (Fig 6C). Decreases in manganese superoxide (MnSOD) expression were seen in HP and HS+HP kidneys at 42 days but not at 7 days (Fig 6D). After 7 days of treatment, catalase expression only decreased in the HS+HP group; however, after 42 days, catalase expression was significantly reduced in the HP, HS, and HS+HP groups (Fig 6E). Seven days of treatment with HP, HS, or both, had no effect on glutathione peroxidase (GPx) expression; however, a severe reduction was observed in the HP, HS and HS+HP kidneys after 42 days (Fig 6F).

**Fig 7:**
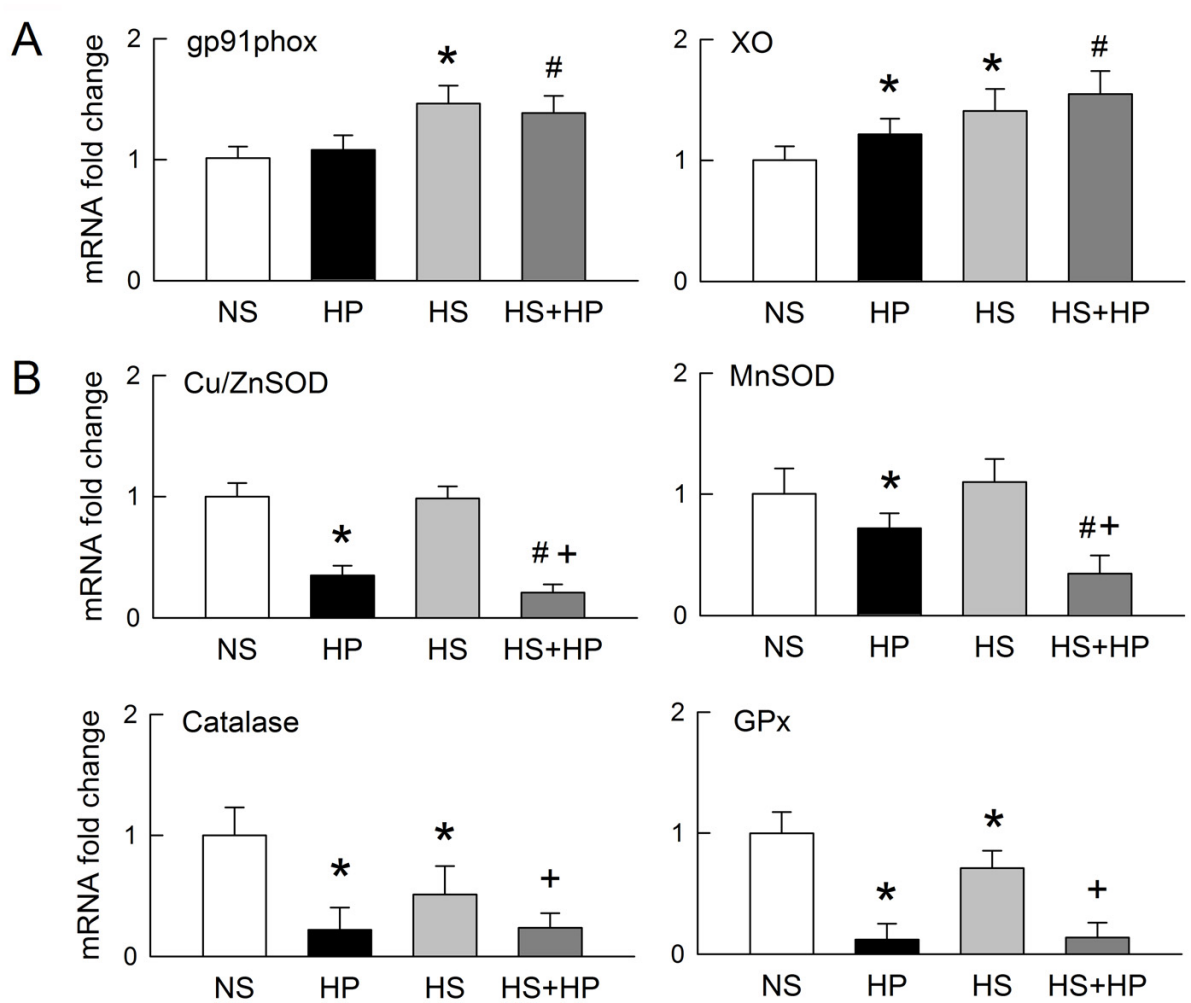
Changes in mRNA expression of redox enzymes in kidneys. Real-time quantitative RT-PCR was used to examine fold changes in the expression of mRNA for NAD(P)H oxidase (as gp91phox), xanthine oxidase (XO) (**A**), and copper and zinc superoxide dismutase (Cu/ZnSOD), manganese superoxide dismutase (MnSOD), catalase, and glutathione peroxidase (GPx) (**B**), in kidneys at 42 days post-induction. ΔC_T_ values were calculated to estimate relative changes in gene expression after subtracting the values for GADPH expression (n = 6 per group). NS, normal sodium; HP, hydroxyl-L-proline; HS, high sodium. *p<0.05, HP or HS groups *vs*. NS group; #p<0.05, HS+HP groups *vs*. HP group; +p<0.05, HS+HP groups *vs*. HS group.

Real-time RT-PCR revealed that most changes in the expression levels of mRNAs for redox enzymes are consistent with their respective protein expression levels (Fig 7). The exception was Cu/ZnSOD mRNA, which was unchanged in HS kidneys after 42 days of treatment (Fig 7B). This discrepancy may be due to post-translational modifications that may affect protein function.

### Changes in oxidative and antioxidant enzyme activity

**Fig 8:**
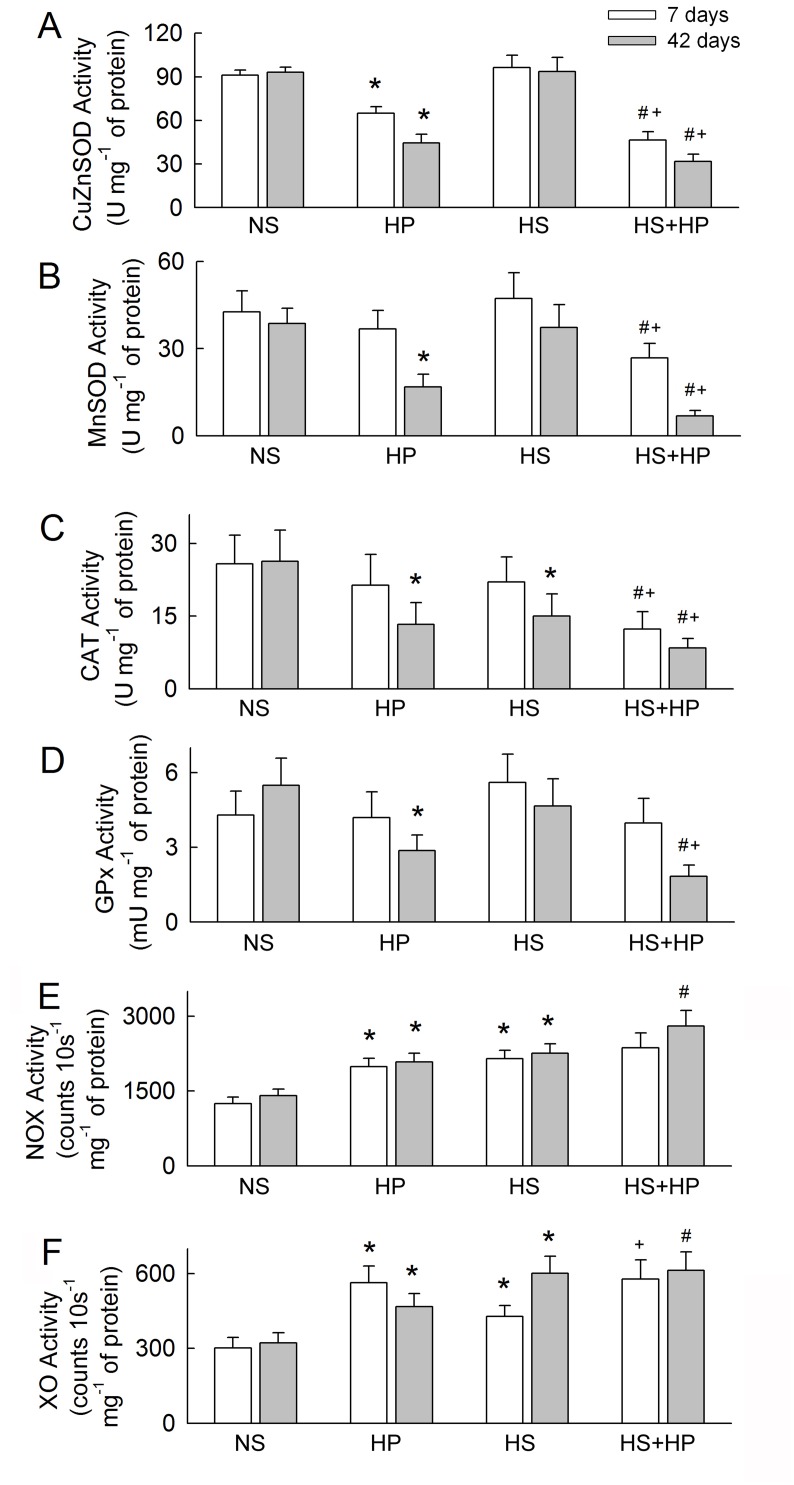
Changes in antioxidant and oxidative enzyme activity. The enzymatic activity of Cu/ZnSOD **(A)**, MnSOD **(B)**, catalase (CAT) **(C)**, GPx **(D)**, NAD(P)H oxidase (NOX) **(E)** and XO **(F)** was determined in whole kidney tissue extracts. N = 6 in each group and time-point. NS, normal sodium; HP, hydroxyl-L-proline; HS, high sodium. *p<0.05, HP or HS *vs*. NS group; #p<0.05, HS+HP *vs*. HP group; +p<0.05, HS+HP *vs*. HS group.

We next examined the activity of antioxidant and oxidative enzymes to strengthen our observations of protein levels. Loss of Cu/ZnSOD activity in the HP-treated kidneys was time-dependent. Most of the reduction was seen after 42 days of treatment, and Cu/ZnSOD activity in the HS+HP kidney was significantly lower than that in the HP kidney (Fig 8A). Only MnSOD activity in the HS+HP kidneys decreased at Day 7 (Fig 8B). MnSOD activity was reduced in the HP and HS+HP kidneys at 42 days. Catalase activity in the HS+HP kidneys decreased at 7 days, and at 42 days in the HP, HS, and HS+HP kidneys, which is consistent with the observed changes in protein levels (Fig 8C). Low GPx activity was observed only in the HP and HS+HP kidneys after 42 days of treatment (Fig 8D). NOX and XO activity was significantly higher in the HP, HS and HS+HP kidneys after 7 and 42 days (Fig 8E and 8F). Compared to the HS or HP kidneys, an additive increase in NOX activity was seen in the HS+HP kidneys at 42 days.

### Changes in anticrystallization protein and citrate transporter expression

**Fig 9:**
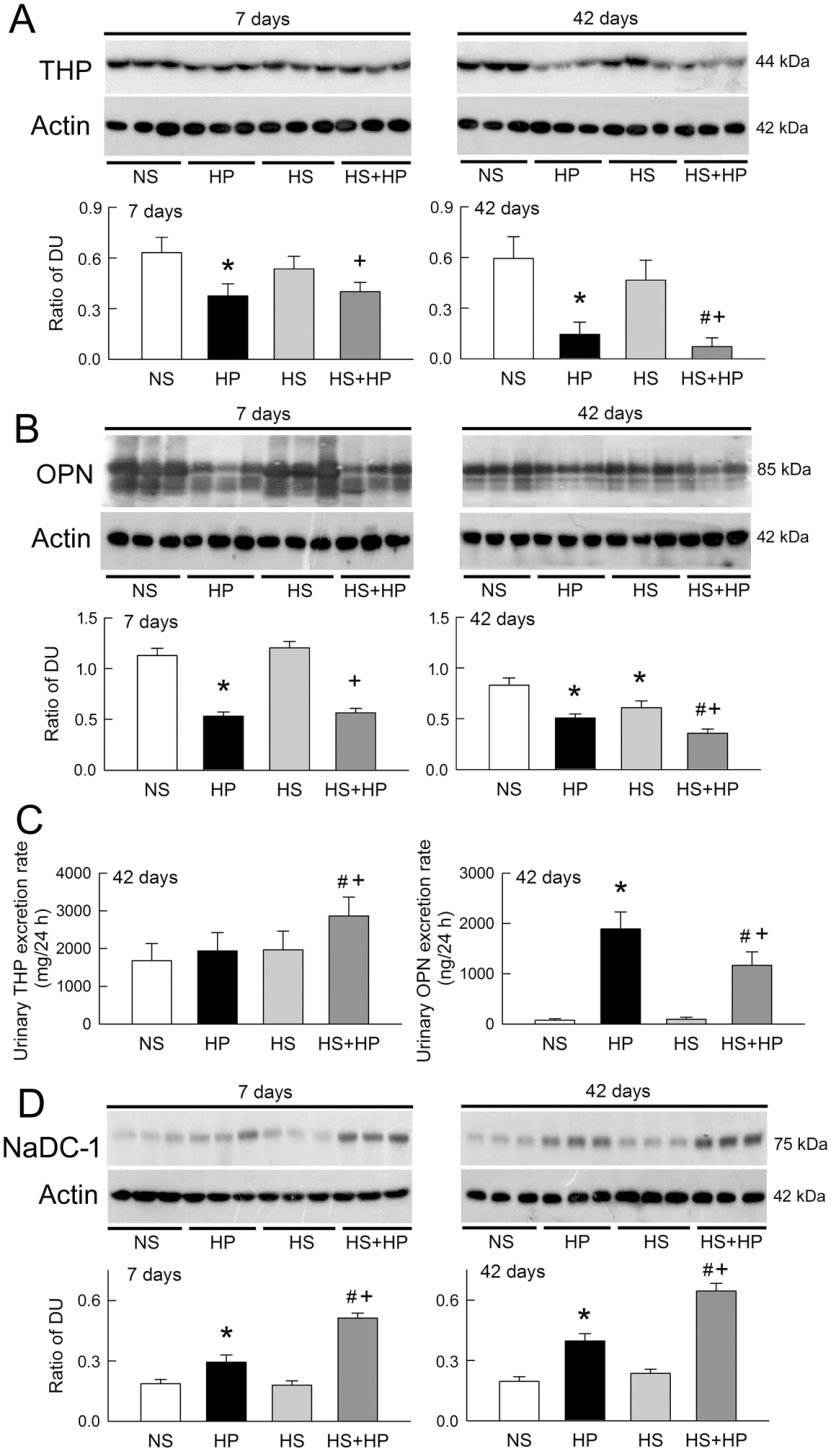
Effect of high sodium and hyperoxaluria on renal anticrystallization defense. Anticrystallization defense was evaluated by assessing the renal or urinary levels of Tamm-Horsfall protein (THP), osteopontin (OPN), and Na^+^-dicarboxylate cotransporter (NaDC-1) expression. Western blots show the renal expression of THP **(A)**, OPN **(B)** and NaDC-1 **(D)** in three representative rats. Equal amounts of protein (40 μg/lane) were loaded. The lower bar graphs show changes in the expression (as measured by densitometry) of THP, OPN, and NaDC-1 normalized to actin. Urinary levels of OPN and THP in rats treated for 42 days were measured by ELISA and expressed as the excretion rate after correction for urine volume. N = 6 in each group and time-point. DU, band density unit; NS, normal sodium; HP, hydroxyl-L-proline; HS, high sodium. *p<0.05, HP or HS *vs*. NS group; #p<0.05, HS+HP *vs*. HP group; +p<0.05, HS+HP *vs*. HS group.

We showed previously that oxidative stress in hyperoxaluric kidneys impairs the anticrystallization defense system [12]. Here we found low THP expression in HP-treated kidneys at 7 and 42 days of treatment (Fig 9A). HS alone only slightly decreased THP expression (the result did not reach statistical significance), but further lowered THP expression in the HS+HP kidney at 42 days. Renal OPN expression decreased in HP-treated rats at both time points (Fig 9B). After 42 days, HS alone reduced renal OPN expression and also acted synergistically with HP. Compared to NS, HP and HS alone did not affect urinary THP excretion; however, a significant increase in urinary THP excretion was observed in HS+HP rats (Fig 9C, left panel). Unlike THP, HP significantly increased urinary OPN excretion after 42 days of treatment (Fig 9C, right panel). HS alone did not affect urinary OPN excretion, but significantly reduced OPN excretion when co-administered with HP.

Citrate transporter NaDC-1 expression increased in the HP and HS+HP kidneys at both time points (Fig 9D). Interestingly, the increases in NaDC-1 expression in the HS+HP kidneys were always higher than those in the HP kidneys. HS itself, however, did not affect NaDC-1 expression when compared to NS.

## Discussion

HS-mediated urinary sodium loss induces calcium loss; the greater the sodium excretion, the greater the loss of calcium [28]. A recent study in rats showed that 8% NaCl for 8 weeks increases urinary calcium excretion 5-fold when compared with 0.3% NaCl [3]. The present study showed that HS alone had no effect on the urinary excretion rate of calcium (Fig 2). A different salt concentration (26.7-fold *vs*. 10-fold compared to the same NS) and induction time for HS treatment may explain this discrepancy. The Δ change in urinary calcium excretion was as follows: HP rats showed a decrease of 38.3±6.6% and 51.7±9.5% compared to NS rats, and HS+HP rats showed a decrease of 32.1±10.4% and 48.5±8.9% compared to HS rats at 7 and 42 days of treatment, respectively. The difference between the groups was not significant, indicating that HP rats and HS+HP rats consume similar amounts of calcium for formation of CaOx crystals. Moreover, low creatinine clearance, seen in the HS+HP rats (Fig 1), impairs glomerular filtration for calcium delivery to excretion. However, HS+HP rats showed more renal deposition and urinary excretion of calcium crystals than HP rats (Fig 2). Therefore, we speculate that factors or resources other than calcium excretion may contribute to the massive crystal formation. An important resource for increasing calcium excretion is the intracellular calcium store. The increased tubular damage caused by exaggerated oxidative stress seen in the HS+HP kidney may be responsible for this disturbance.

A previous study demonstrated that 7 days of HS intake increases oxidative stress via an increase in NOX and a decrease in Cu/ZnSOD and MnSOD expression at the mRNA level; these phenomena were associated with increased urinary excretion of the lipid peroxidation metabolites, MDA and 8-isoprostane F_2α_ [9]. Here, we also showed that HS increases urinary MDA excretion, an effect that persisted at 42 days (Fig 4). In addition to NOX and SODs, the present results also showed increased XO expression and decreased CAT and GPx protein and mRNA expression in HS kidneys, with changes in enzyme activity in some cases (Figs 6–8). Contrary to the previous findings, Dobrian *et al*. reported that a 10 week HS diet had no effect on urinary levels of 8-isoprostane F_2α_ in control rats [29]. They, however, also demonstrated increases in superoxide anion formation in aortic tissues [29]. Using *in situ* superoxide staining, the present study showed increased superoxide formation in the HS kidney (Fig 5), which strongly supports the presence of oxidative stress in renal tissues. We conclude that HS disrupts the homeostatic oxidative/antioxidant balance and then increases oxidative stress in the kidneys. What would be the consequence of this disruption? Our results showed that HS triggers apoptotic injury, as evidenced by increases in cytochrome c release and PARP activity; this injury, however, was not severe enough to induce tubular damage (Fig 4). When HS was introduced to the hyperoxaluric kidney, additive effects on oxidative stress, tubular damage, renal function impairment, and massive crystal formation were observed. Similar to our previous findings, hyperoxaluric rats fed a low-vitamin E diet also showed enhanced intrarenal redox imbalance caused by depletion of antioxidant protein defense and increased oxidative protein activity, leading to more renal damage and massive CaOx crystal deposition [23].

In addition to exaggerated oxidative stress, the present study demonstrated loss of anticrystallization defenses, such as hypocitraturia, hypomagnesuria, and low renal OPN and THP expression, in HP kidneys (Figs 2 and 9). These results are consistent with those of previous reports [12, 19, 30] and clearly indicate deficient defense against CaOx formation in the hyperoxaluric kidney. Hypocitraturia and loss of THP and OPN were worse in HS+HP kidneys, especially at 42 days of treatment, than in HP kidneys (Figs 2 and 9). Citrate can be synthesized by cytoplasmic ATP citrate lyases and mitochondrial aconitases, and is freely filtered in the glomerulus and reabsorbed by NaDC-1 in the proximal tubule [31]. Although we did not examine *in situ* renal NaDC-1 expression, upregulation favored more citrate reabsorption and led to hypocitraturia in hyperoxaluric rats with or without HS (Fig 2). A previous study showed that chronic metabolic acidosis leads to an increase in rat renal cortical NaDC-1 mRNA and protein expression in the apical membrane of proximal tubules [32]. Therefore, we considered whether metabolic acidosis in hyperoxaluric rats may account for increases in renal NaDC-1 expression (Fig 9); however, acidemia and low urinary pH (due to increased acid excretion) were not seen in HP or HS+HP rats (data not shown). Although the urine pH of HP-treated rats was lower than that of the controls, the differences were not significant (data not shown). Hence, metabolic acidosis seems to not be the mechanism underlying NaDC-1 upregulation in hyperoxaluric kidneys. So what would cause hypocitraturia observed in this study? The present results and our previous data showed that nephrolithiasis includes oxidative stress [10, 12, 23, 29]. Exaggerated oxidative stress associated with lower hypocitraturia was found when hyperoxaluric kidneys were treated with HS (Figs 2–8). Since increased cellular levels of reactive oxygen species (ROS), as evidenced by the increased superoxide generation (Fig 5), regulate gene expression, we speculate that hyperoxaluria-induced NaDC-1 upregulation may be mediated by ROS. However, this hypothesis remains unproven. Compared with the NS kidney, the HS kidney showed evidence of excess superoxide formation and dysregulation of antioxidant/oxidant expression and activity (Figs 5–8); however, there were no associated changes in NaDC-1 expression (Fig 9D). Moreover, the present results cannot rule out the possible intracellular effect of acidosis on the regulation of NaDC-1 expression in the tubular epithelium.

OPN and THP are two important factors in the kidney that not only prevent CaOx crystal formation but also provide renoprotection [30]. After treatment with phenylephrine, rats fed a HS diet show hypertension associated with *de novo* synthesis of OPN in the kidneys [33]. By contrast, OPN expression in the tubules was decreased in rats fed a SD diet [34]. These results indicate that HS increases, and low salt decreases, OPN expression. Contrary to these findings, the present study showed that HS decreases OPN expression in hyperoxaluric kidneys more than in control kidneys (Fig 9). This suggests that hyperoxaluria itself has a more profound effect on OPN expression than HS; however, HS still synergized with hyperoxaluria to lower renal OPN expression (Fig 9B). A previous study of urolithiasis showed that urinary OPN concentrations in patients with no stone were significantly higher than those in urolithiasis patients with a tendency toward stone enlargement [35]. Our animal model demonstrated that HS+HP rats showed increased urinary OPN excretion, but that this increase was smaller than that in HP rats at 42 days (Fig 9C). Meanwhile, HS induced greater excretion of CaOx crystals in urine in hyperoxaluric kidneys than in control kidneys (Fig 2). We speculate that more urinary OPN is consumed in HS+HP rats than in HP rats to prevent crystal growth and to increase crystal excretion.

A previous study in THP knockout mice revealed spontaneous formation of calcium crystals in the kidneys, and dramatic increases in both the frequency and the severity of crystal formation, when animals were fed excessive calcium and oxalate [19]. Similarly, our results showed that renal THP expression is reduced in hyperoxaluric kidneys that contain CaOx crystals, indicating that THP deficiency could be an important contributing factor to crystal formation. Interestingly, HS alone also reduced renal THP expression at 42 days; however, we found no spontaneous crystal formation in HS kidneys. The degree of THP reduction may explain these differences. Moreover, our results showed no increase in urinary THP excretion in the HS group after 42 days of treatment (Fig 9C). This is consistent with a previous study that demonstrated similar urinary levels of THP in healthy, but genetically hypertension-prone, subjects before and after HS treatment [36]. Urinary THP excretion, however, increased in HS+HP rats (Fig 9C). We did not explore the mechanism underlying this observation. However, a previous study demonstrated that increases in superoxide formation during HS diet causes oxidative stress in the renal medulla [9]. Since it is secreted by the thick ascending limb of Henle’s loop, which is involved in the regulation of salt excretion [34], we speculate that increased THP excretion reflects the increased sensitivity of the thick ascending limb in hyperoxaluric kidneys to HS-induced oxidative stress.

In conclusion, HS diet aggravates hyperoxaluria-induced renal damage and CaOx crystal formation. The underlying mechanism appears to be exacerbation of oxidative and antioxidant imbalance, which is already present in the hyperoxaluric kidney. Exaggerated oxidative stress in HS-treated hyperoxaluric kidneys possibly increases citrate transporter NaDC-1 expression and lowers OPN and THP defense, leading to a worsening of hypocitraturia and poor anticrystallization against CaOx crystal formation.
